# Monitoring prognosis in severe traumatic brain injury

**DOI:** 10.1186/cc13915

**Published:** 2014-06-11

**Authors:** Andrew IR Maas, Ewout W Steyerberg

**Affiliations:** 1 Department of Neurosurgery, Antwerp University Hospital and University of Antwerp, Wilrijkstraat 10, 2650 Edegem, Belgium; 2 Department of Public Health, Erasmus MC - University Medical Center Rotterdam, PO Box 2040, Rotterdam, CA 3000, Netherlands

## Abstract

The choice of disease-specific versus generic scales is common to many fields of medicine. In the area of traumatic brain injury, evidence is coming forward that disease-specific prognostic models and disease-specific scoring systems are preferable in the intensive care setting. In monitoring prognosis, the use of a calibration belt in validation studies potentially provides accurate and intuitively attractive insight into performance. This approach deserves further empirical evaluation of its added value as well as its limitations.

## 

In the previous issue of *Critical Care*, Raj and colleagues [1] report a detailed study on the evaluation of commonly employed general ICU scales to predict outcome in patients with traumatic brain injury (TBI). They compare performance to that of simpler models based on only age and Glasgow Coma Scale (GCS). The authors conclude that the simple prognostic model based only on age and GCS showed fairly good prognostic performance and that the use of more complex general ICU scoring systems added little to this. This manuscript clearly demonstrates that TBI patients in the ICU environment are a highly specific population, in which general ICU scoring systems are of limited value. Second, from a methodological perspective, it presents and discusses essential approaches for quantifying the performance of prognostic models and provides empirical illustration of the use of a new instrument for assessing calibration.

## Limited value of general ICU scoring systems in traumatic brain injury patients

Scoring systems such as the Acute Physiology and Chronic Health Evaluation II, Simplified Acute Physiology Score II and Sequential Organ Failure Assessment scores are commonly used in the intensive care setting to quantify the impact of disease severity and to benchmark the quality of delivered health care. These scoring systems are developed for use in the general ICU environment and are not disease specific. TBI is a very heterogeneous disease in terms of cause, pathology, severity and also in expected outcome. Disease-specific prognostic models for moderate and severe TBI have been developed and validated. These include the CRASH (Corticosteroid Randomisation After Significant Head Injury) [2] and the IMPACT (International Mission for Prognosis and Analysis of Clinical Trials in TBI) [3] prognostic models. Both the IMPACT and CRASH models have shown reasonable to good performance at external validation, both for mortality and 6-month Glasgow Outcome Scale. The latter is particularly important as the degree of functional recovery in the long term is perhaps even more relevant than early mortality in TBI patients. The IMPACT studies have shown that most prognostic information is contained within three variables: age, GCS motor score, and pupillary reactivity [4]. The findings on age and GCS in the present study are in line with this observation. Apparently the additional information from many parameters obtained at admission (as in the IMPACT model) or during the first 24 hours of care (as for ICU-specific models) adds little prognostic value compared to core information such as age and admission GCS. Further development and validation of TBI-specific prediction models is required, including disease-specific information that becomes available during the clinical course. The latter will require a dynamic prediction framework [5].

## Measuring performance of prognostic models in the traumatic brain injury population

The performance of prognostic models is commonly evaluated by discrimination and calibration. Discrimination concerns the ability to distinguish between survival and death or favourable and unfavourable outcome. It is generally assessed by calculating the area under the receiver operating characteristic curve (AUC). Discrimination is influenced by both the validity of the model for a specific population (that is, the statistical fit of the model) and the case mix of the validation population [6,7]. If the population includes subsets with a more extreme prognosis (for example, mild versus severe TBI), the discriminative ability will be boosted upwards. For this reason, a case mix adjusted AUC has been proposed [6]. In the present study, a more homogeneous population may have lowered the discriminative ability of the ICU-specific models.

Calibration evaluates the agreement between observed and predicted outcome and can be graphically presented in plots. The often used Hosmer-Lemeshow test considers deciles of patients with similar risk, and reflects the average concordance of expected outcome compared to the observed outcome analyzed. Limitations of the Hosmer-Lemeshow test mentioned by the authors include that it may be non-informative in large data sets (that is, statistically significant for minor miscalibration), and that the division of the patient cohort into deciles does not account sufficiently for the individual patient.

As a relatively new instrument to assess calibration, the authors utilised a calibration belt. This approach was developed and tested within the GiViTI consortium in Italy (Italian Group for the evaluation of interventions in intensive care medicine) and was taken forward in a larger ICU network named Prosafe through EU funding (PHEA 2007 331). These studies are now coordinated in the CREACTIVE Project (prospective longitudinal data collection and comparative effectiveness research for TBI). Within this project, TBI-specific prognostic models will be developed to be used as a benchmark for quality of care assessment in individual ICUs. The calibration belt relates the observed and the expected probability of a dichotomised outcome. Importantly, the calibration belt calculates the 80% confidence interval and the 95% confidence interval surrounding the calibration curve. This instrument thus potentially provides accurate and intuitively attractive insight into calibration performance. As with any new instrument, however, its validity has to be demonstrated in broad settings and validated by other groups. Limitations may only become apparent with greater experience. It is not quite clear how dependent the calibration belt and, in particular, the calculated confidence interval may be upon the relative number of patients with specific prognostic risks. Adding the distribution of patient numbers across the plotted curves would provide additional insight. Notwithstanding this potential limitation, this approach in which disease-specific aspects are combined in an intuitively attractive novel instrument is worthy of further exploration and validation.

## Abbreviations

AUC: Area under the receiver operating characteristic curve; CRASH: Corticosteroid randomisation after significant head injury; GCS: Glasgow coma scale; IMPACT: International mission for prognosis and analysis of clinical trials in TBI; TBI: Traumatic train injury.

## Competing interests

The authors declare that they have no competing interests.
